# Data-Driven Analysis of EEG Reveals Concomitant Superficial Sleep During Deep Sleep in Insomnia Disorder

**DOI:** 10.3389/fnins.2019.00598

**Published:** 2019-07-09

**Authors:** Julie Anja Engelhard Christensen, Rick Wassing, Yishul Wei, Jennifer R. Ramautar, Oti Lakbila-Kamal, Poul Jørgen Jennum, Eus J. W. Van Someren

**Affiliations:** ^1^Danish Center for Sleep Medicine, Department of Clinical Neurophysiology, Rigshospitalet Glostrup, Glostrup, Denmark; ^2^Department of Health Technology, Technical University of Denmark, Kongens Lyngby, Denmark; ^3^Department of Sleep and Cognition, Netherlands Institute for Neuroscience, an Institute of the Royal Netherlands Academy of Arts and Sciences, Amsterdam, Netherlands; ^4^Department of Integrative Neurophysiology, Center for Neurogenomics and Cognitive Research (CNCR), Amsterdam Neuroscience, VU University Amsterdam, Amsterdam, Netherlands; ^5^Amsterdam UMC, Vrije Universiteit, Psychiatry, Amsterdam Neuroscience, Amsterdam, Netherlands

**Keywords:** insomnia, indiscrete labeling of sleep, vigilance states, topic modeling, data-driven analysis, polysomnography, latent Dirichlet allocation

## Abstract

**Study Objectives:** The subjective suffering of people with Insomnia Disorder (ID) is insufficiently accounted for by traditional sleep classification, which presumes a strict sequential occurrence of global brain states. Recent studies challenged this presumption by showing concurrent sleep- and wake-type neuronal activity. We hypothesized enhanced co-occurrence of diverging EEG vigilance signatures during sleep in ID.

**Methods:** Electroencephalography (EEG) in 55 cases with ID and 64 controls without sleep complaints was subjected to a Latent Dirichlet Allocation topic model describing each 30 s epoch as a mixture of six vigilance states called Topics (T), ranked from N3-related T1 and T2 to wakefulness-related T6. For each stable epoch we determined topic dominance (the probability of the most likely topic), topic co-occurrence (the probability of the remaining topics), and epoch-to-epoch transition probabilities.

**Results:** In stable epochs where the N1-related T4 was dominant, T4 was more dominant in ID than in controls, and patients showed an almost doubled co-occurrence of T4 *during* epochs where the N3-related T1 was dominant. Furthermore, patients had a higher probability of switching from T1- to T4-dominated epochs, at the cost of switching to N3-related T2-dominated epochs, and a higher probability of switching from N2-related T3- to wakefulness-related T6-dominated epochs.

**Conclusion:** Even during their deepest sleep, the EEG of people with ID express more N1-related vigilance signatures than good sleepers do. People with ID are moreover more likely to switch from deep to light sleep and from N2 sleep to wakefulness. The findings suggest that hyperarousal never rests in ID.

## Statement of Significance

Insomnia Disorder (ID) is the most prevalent sleep disorder. It is poorly understood why people with ID experience part of their sleep as being awake. Quantitative EEG analyses may aid to solve the impasse. We used symbolic representations of spectral changes within 3 s windows to describe standard 30 s sleep epochs as a mixture of states. The method revealed that, as compared to controls, people with ID experience twice as much concurrent light sleep during the deepest sleep, suggesting that hyperarousal continues during deep sleep. Future studies could address the value of concurrent light sleep as a biomarker to pursue brain mechanisms involved in ID and understand treatment response variability.

## Introduction

Insomnia disorder (ID) is characterized by persistent difficulty initiating or maintaining sleep associated with daytime dysfunction which cannot be attributed to insufficient opportunity for sleep (American Academy of Sleep Medicine, 2014). More than 6% of the adult population in high-income countries suffer from chronic insomnia, and this number increases to almost 50% when including the acute form of insomnia (Ohayon, 2002). Moreover, ID has been identified as the second most common mental disorder in Europe (Wittchen et al., 2011). Only recently, genome-wide association studies have commenced to reveal biological pathways involved in insomnia (Hammerschlag et al., 2017; Jansen et al., 2019). Phenotypically, ID can be characterized as a persistent state of physiological and psychological hyperarousal, resembling the state normally seen only transiently during stress (Bonnet and Arand, 2010; Riemann et al., 2010). Polysomnography (PSG) findings in subjects with ID include reductions in sleep continuity and in the time spent in slow-wave sleep and rapid-eye-movement (REM) sleep (Baglioni et al., 2014). These reductions, however, hardly correlate with the subjective severity of sleep complaints (Manconi et al., 2010) in ID and typically underestimate it. The question may be raised whether the common PSG variables capture the key neurophysiological impairments in ID.

More recent methods go beyond the standard manual PSG scoring and give a more detailed description of sleep in ID. An important aim of these methods is to close the present gap between the objectively recorded sleep and the subjective experience of ID (Feige et al., 2013). A recent review revealed promising use of auditory stimulation and event-related potential (ERP) recordings for understanding the mismatch between subjective and objective sleep quality and quantity (Bastien et al., 2014). This technique may not readily be available in routine clinical PSG assessment. To quantify intrinsic rather than responsive processes, most methods focus on the analysis of ongoing sleep EEG. A first class of methods utilizes measures of the spectral composition of the EEG signal. The most robust finding may be that the EEG-spectrum of people with ID contains more high frequency power during pre-sleep wakefulness (Freedman, 1986; Colombo et al., 2016a), and during non-REM (NREM) sleep (Israel et al., 2012; Spiegelhalder et al., 2012; Maes et al., 2014), which may be involved in the degree of perceiving sleep as wakefulness (Krystal et al., 2002). A second class of methods focuses on characterization of transient event occurrence. For example, the REM sleep instability model of ID (Riemann et al., 2012) states that micro- and macro-arousals occur more frequently during and around REM sleep; these arousals have been proposed to be involved in more thought-like cognitions (Wassing et al., 2016). Findings on spindles and K-complexes are equivocal. Studies reported either a decreased spindle density in ID (Besset et al., 1998) or no change (Bastien et al., 2009a). K-complex density was reported to be increased (Forget et al., 2011) or normal (Bastien et al., 2009b).

A third class of methods focusses on the temporal dynamics in the EEG signal. Most analyses of the cyclic alternating pattern (CAP) agree that people with ID express an increased CAP rate (Terzano et al., 2003; Chouvarda et al., 2011, 2012), suggested to indicate “destabilization” of sleep in people with ID (Parrino et al., 2004), which may be improved by certain hypnotics (Parrino et al., 2004, 2008; Ozone et al., 2008). One study further concludes that there is a link between CAP phase A2 subtype and sleep state misperception in ID (Parrino et al., 2009). In a recent study, 30 s epochs of staged sleep were regarded as a Markov chain dynamical process with individual-specific probabilities to switch from one state to another (Wei et al., 2017). It was shown that the probability of transitioning from N2 sleep to N1 sleep or wakefulness is most consistently increased in ID. In another approach, stronger long-range temporal correlations in EEG power fluctuations during pre-sleep wakefulness were associated with worse subjective sleep quality within ID and within normal sleepers (Colombo et al., 2016b). In conclusion, it seems that the association between the subjective complaints and objective sleep parameters may be better revealed when the brain activity assessed with EEG is considered as a complex dynamical process.

A consideration when searching for objective measures that better capture subjective complaints is that traditional sleep classifications all presume a strict sequential occurrence of global brain states. Recent studies challenged this presumption by showing evidence for concurrent sleep- and wake-type neuronal activity (Nir et al., 2011; Vyazovskiy et al., 2011; Funk et al., 2016). Thus, ideally, novel objective measures to pursue neural correlates of subjective sleep complaints in insomnia should not be limited to quantifying vigilance states sequentially, but also quantify their concurrency.

Based on these considerations, we here analyzed the sleep EEG of people with ID and controls without sleep complaints, using a data-driven topic model previously developed and validated in our lab (Koch et al., 2014; Christensen et al., 2016). Originally, topic modeling is a probabilistic approach used to reveal underlying themes or topics in text documents, based on the presence and combination of words (Blei et al., 2003). The same method has been employed to describe the proportions of different underlying latent vigilance states that are simultaneously present in each 30 s sleep epoch. Latent vigilance states are determined in a data-driven way based on symbolically represented patterns observed in the EEG power spectrum. Every epoch can be described as a mixture of six topics T (T1-T6), each related but not equal to a classical sleep stage. The topics T can be ranked from N3-related T1 and T2 to wakefulness-related T6. The topic modeling approach for analyzing sleep EEG has several advantages: (1) It includes high-dimensional information that is lost with manual scoring, (2) it outputs a mixture of states rather than a discrete scoring, and (3) epochs are not biased by subjective interpretation or classification of neighboring epochs. Lastly, although the symbolic representation is based on scaled EEG and EOG spectral measures, this method goes beyond standard spectral analysis methods and analysis of specific micro-sleep phenomena. The method captures latent patterns in each epoch and visualizes this into a graphical two-dimensional representation. It captures latent patterns in each epoch that indicate the sequential occurrence of different vigilance states, but also captures latent patterns that are indicative of several concomitant vigilance states. An important note here is that EEG leads integrate neurophysiological activity across tens- to hundreds of thousands of neurons. Therefore, the seemingly temporal simultaneous vigilance states observed at the level of the scalp, may well be due to spatial differences in vigilance states of small groups of neurons. We use the term “concomitant” to indicate both simultaneous and sequential occurrences of different vigilance states.

By comparing the sleep EEG of people with ID and controls in this data-driven way, we aimed to reveal group differences that might not be detectable by current methods. For instance, local wakefulness intrusions in sleep would imply simultaneous wake- and sleep EEG signatures. We therefore hypothesized a stronger dominance and co-occurrence of wakefulness-related topics in people suffering from ID than in controls without sleep complaints. We moreover expected that the sleep EEG of people with ID would have higher probability to transition from epochs dominated by deeper sleep-related topics to epochs dominated by light sleep-related topics.

## Materials and Methods

### Participants

Participants were recruited through advertisement and the Netherlands Sleep Registry website (NSR^1^) (Benjamins et al., 2017). A total of 55 participants with ID (41 females, 47.8 ± 12.9 years) and 64 controls (42 females, 45.3 ± 14.6 years) were included. The study was approved by the ethics committee of the Amsterdam University Medical Center, Amsterdam, Netherlands. All participants provided written informed consent. Inclusion criteria for ID were in accordance with the Diagnostic and Statistical Manual of Mental Disorders, fifth edition (American Psychiatric Association, 2013). Exclusion criteria for controls were any sleep difficulties. Exclusion criteria for all participants were neurological, psychiatric, somatic conditions, or other diagnosed sleep disorders including sleep apnea, restless legs syndrome, narcolepsy, or circadian rhythm disorder. In addition, no sleep medication in the previous two months was allowed. Demographic and PSG variables are summarized in Table 1.

**Table 1:** Demographics and sleep characteristics (subjective, AASM-based, and LDA model-based) in ID and controls.

	Controls	ID	P
*Demographics*			
N	64	55	-
Age [years, μ ± σ]	45.3 ± 14.6	47.8 ± 12.9	0.41
Sex [male/female]	22/42	14/41	0.29
*Subjective insomnia*		
**ISI [μ ± σ]**	**3.8 ± 3.7**	**16.5 ± 4.3**	**<0.0001**
*AASM-based measures*		
TiB [min, μ ± σ]	483.3 ± 52.7	471.6 ± 44.5	0.10
**TST [min, μ ± σ]**	**421.7 ± 60.7**	**400.6 ± 59.3**	**0.03**
SE [%, μ ± σ]	87.3 ± 8.6	85.1 ± 10.7	0.32
Wake [%, μ ± σ]	11.7 ± 8.7	14.3 ± 10.9	0.23
REM [%, μ ± σ]	20.2 ± 6.7	19.4 ± 8.5	0.45
N1 [%, μ ± σ]	3.6 ± 2.4	5.0 ± 4.1	0.13
N2 [%, μ ± σ]	39.9 ± 9.4	37.8 ± 11.1	0.12
N3 [%, μ ± σ]	23.6 ± 9.0	22.9 ± 10.2	0.62
*LDA model-based overall average topic probability*		
T1 [%, μ ± σ]	5.69 ± 1.67	5.23 ± 1.50	0.23
T2 [%, μ ± σ]	25.06 ± 2.64	24.86 ± 2.65	0.52
T3 [%, μ ± σ]	31.08 ± 3.14	31.70 ± 2.77	0.44
T4 [%, μ ± σ]	12.81 ± 1.43	13.08 ± 1.38	0.26
T5 [%, μ ± σ]	18.97 ± 2.88	18.76 ± 2.63	0.94
T6 [%, μ ± σ]	5.62 ± 1.66	5.59 ± 1.42	0.56
*LDA model-based percentage of time of stable epochs for each stable epoch type and in total (for the subset of participants with a non-zero number of stable epochs)*		
Stable T1 epochs [%, μ ± σ] (number of subjects expressing non-zero values)	1.51 ± 2.09 (N_C_ = 30)	1.11 ± 1.24 (N_ID_ = 22)	0.57
Stable T2 epochs [%, μ ± σ] (number of subjects expressing non-zero values)	19.98 ± 4.71 (N_C_ = 64)	20.01 ± 4.51 (N_ID_ = 55)	0.94
Stable T3 epochs [%, μ ± σ] (number of subjects expressing non-zero values)	24.33 ± 6.04 (N_C_ = 64)	24.72 ± 5.38 (N_ID_ = 55)	0.98
Stable T4 epochs [%, μ ± σ] (number of subjects expressing non-zero values)	2.73 ± 2.44 (N_C_ = 62)	3.42 ± 2.83 (N_ID_ = 55)	0.24
Stable T5 epochs [%, μ ± σ] (number of subjects expressing non-zero values)	7.33 ± 4.43 (N_C_ = 64)	7.67 ± 4.54 (N_ID_ = 55)	0.46
Stable T6 epochs [%, μ ± σ] (number of subjects expressing non-zero values)	1.59 ± 1.94 (N_C_ = 44)	1.06 ± 1.26 (N_ID_ = 37)	0.34
Stable epochs in total [%, μ ± σ] (number of subjects expressing non-zero values)	56.08 ± 5.42 (N_C_ = 64)	57.29 ± 6.59 (N_ID_ = 55)	0.23

*The P-value for the statistics on sex is obtained from a Chi-square test. All other P-values were obtained from Wilcoxon rank sum tests. Bold font-style indicates significant group differences. ID, insomnia disorder; ISI, insomnia severity index; TiB, time in bed from lights out time to get up time; TST, total sleep time; SE, sleep efficiency; T1-T6, topic 1 to 6; LDA, latent Dirichlet allocation*.

### Procedure and EEG Recording

Participants were instructed to maintain a regular sleep/wake schedule during 1 week prior to laboratory assessment. On the days of laboratory assessments, they were furthermore instructed to refrain from alcohol and drugs and to limit their caffeinated beverages to a maximum of two cups before 12:00 noon. EEG was recorded for two consecutive nights between 11:00 PM and 7:00 AM using a 256-electrode HydroCel net connected to a Net Amps 300 amplifier (Electrical Geodesic Inc. (EGI), Eugene, OR, United States; input impedance: 200 MΩ, A/D converter: 24 bits). Electrode impedance was kept below 100 kΩ. Signals were acquired with a sampling frequency of 1,000 Hz and referenced to the Cz electrode. Sleep-stage scoring was performed in accordance to the American Academy of Sleep Medicine (AASM) standard (Iber et al., 2007). Data from the second night of sleep were analyzed.

### EEG Preprocessing

Pre-processing steps were performed using the MEEGPIPE toolbox^2^ and EEGLAB (Delorme and Makeig, 2004) in Matlab R2014a (The Mathworks Inc., Natick, MA, United States). First, signals were downsampled to 250 Hz with an antialiasing low-pass filter at 80 Hz. Because the original analysis procedure (Koch et al., 2014) was developed and validated on the derivations C3-A2, O1-A2, EOGL-A2, and EOGR-A1, we analyzed the rereferenced signal between those electrode pairs. Visual inspection of data quality employed “EEG viewer,” a Matlab based software developed by Miki Nikolic at Danish Center for Sleep Medicine at Rigshospitalet, Glostrup, Denmark (DCSM). In case the signal was of poor quality, a neighboring electrode was selected.

Signals were filtered forward and reverse in time using 4th order Butterworth filters with cutoffs (-3 dB) at 0.3 and 35 Hz for the EEG signals, and 0.3 and 10 Hz for the EOG signals. All epochs between lights off and lights on were analyzed except for epochs in the beginning or end of the night that were visually clearly contaminated by artifacts. The signal quality of each remaining epoch was evaluated using the standard deviation and range of the signal, and the range of the first derivative of the signal. An epoch was rejected in case the epoch-wise mean of any of the three statistics surpasses four median absolute derivations, as previously described in the supplementary materials of Colombo et al. (2016a). Epochs with artifacts were omitted from analysis.

### Sleep Analysis Procedure

**Figure 1:**
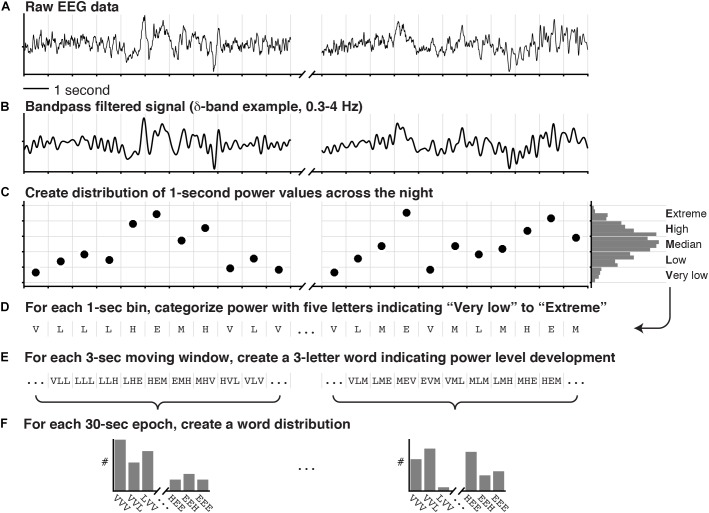
Symbolization of raw EEG for the purpose of Latent Dirichlet Allocation (LDA) topic modeling. The raw data of two EEG signals of which one is depicted in **(A)**, were bandpass filtered into the classical frequency bands (δ, θ, α, and β). **(B)** One bandpass filtered signal is shown. **(C)** The power in each 1 s window was calculated and summarized in a distribution across the entire recording. **(D)** According to quintiles of this distribution, the power in each 1 s window was categorized as either “**V**ery low,” “**L**ow,” “**M**edian,” “**H**igh,” or “**E**xtreme,” effectively creating a vector of letters. **(E)** For each 3 s sliding window, three consecutive letters were concatenated to create words indicating the spectral power-level development. **(F)** Finally, a word distribution was created for each 30 s epoch by counting how often each of the possible words occurred (5 categories and 3 letters: 5^3^ = 125 words per frequency-band per EEG channel). In addition to the EEG words, an additional 192 words were created in a similar fashion [4 categories and 3 letters: 4^3^ = 64 words per EOG signal (left and right) plus 64 words for the cross-correlation between the EOG signals].

**Figure 2:**
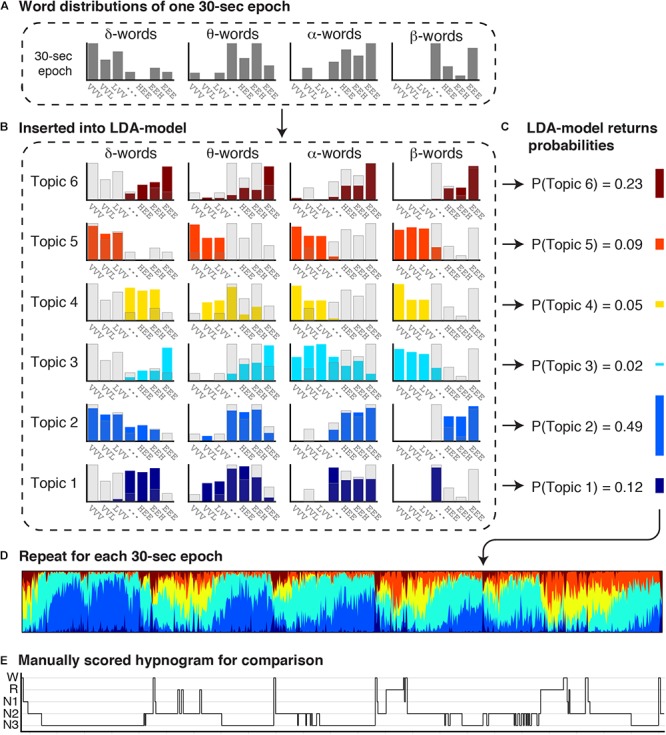
Estimation of concurrent vigilance states in each 30 s epoch. The combined word distributions for each 30 s epoch were inserted into a Latent Dirichlet Allocation (LDA) model, which returns a mixture of topic probabilities for each epoch. The LDA topic model was previously trained to learn the particular distribution of words for six topics (Koch et al., 2014). **(A)** The observed word distributions of one 30 s epoch is inserted into the LDA topic model. **(B)** The *observed* word distributions of the 30 s epoch are depicted in transparent gray bars, and the *expected* word distributions for each topic in colored bars. **(C)** By comparing the observed and expected word distributions, the LDA topic model returns the probability that the observed word distributions comes from the word distribution of each topic. Each 30 s epoch is thereby represented as a mixture of six topics. **(D)** A topic diagram obtained by repeating this procedure for each 30 s epoch in the recording. Each vertical bin in the topic diagram is a mixture of six colors, where the hight of each stacked color represents the probability of a topic. Colors codes range from dark blue (T1) to red (T6). **(E)** For comparison, the manual scored hypnogram is presented below the topic-diagram.

Artifact-free epochs were analyzed using a validated automated topic modeling procedure (Koch et al., 2014). As described above, based on the occurrence and combination of symbolic representations of spectral patterns in the raw EEG data, the topic model describes each epoch as a mixture of vigilance states rather than as a single sleep stage. Latent Dirichlet Allocation (LDA) topic modeling is a machine learning method, traditionally used on text documents to reveal the topics based on the presence and combination of words in the document (Blei et al., 2003). In a similar way, we here used the LDA model procedure to reveal underlying vigilance states (*topics*) based on the occurrence of spectral patterns (*words*). Figure 1 illustrates the procedure of symbolizing the raw EEG and building distributions of spectral patterns denoted as “word” distributions. Figure 2 illustrates how the LDA model used the “word” distribution within an epoch to estimate the probabilities of latent vigilance states (*topics*) for that epoch.

#### Symbolization of the Raw EEG

First, we performed fast-fourier spectral analysis on each 1 s bin of the recording to obtain a participant-specific distribution of spectral power within each classical frequency band. Five equally sized categories were defined by the quintiles of these distributions (*Extreme*, *High*, *Median*, *Low*, or *Very low* power; Figure 1A–C). Secondly, each 1 s segment was categorized according to these quintile cutoffs (Figure 1D), effectively generating a long vector of letters (*E*, *H*, *M*, *L*, or *V*) for each of the classical frequency bands. Then, for each frequency band, 28 3-letter *words* were generated for each 30 s epoch by concatenating the letters from moving 3 s windows with steps of 1 s. Each 3-letter word descibes a specific pattern of change in spectral power (e.g., HLM: high-low-median; Figure 1E). Lastly, we counted how often each possible word occurred in the 30 s epoch, creating a word distribution (Figure 1F). Note that for two EEG channels (C3-A2 and O1-A2) the word distributions were created for each classical frequency band (δ, θ, α, and β), and for each EOG channel (EOGL-A2 and EOGR-A1) one word distribution was created for the spectral power below 5 Hz, and one for the cross-correlation values between the two EOG channels instead of power (Koch et al., 2014).

#### LDA Probabilistic Topic Modeling

Figure 2 illustrates how the LDA model combines word distributions across the EEG and EOG channels to estimate the probabilty of occurence of six vigilance states (*topics*) within each epoch. The LDA model has previously been trained and validated on an independent dataset (Koch et al., 2014). In short, we applied a machine learning approach for non-linear classification of more that two classes (multiclass support vector machine using a radial basis-function kernel) to find that classification in six different topics (rather than 3, 4, 5, or 7) resulted in the highest accuracy for the LDA model output to replicate traditional PSG-scoring. Then we trained the LDA model, where it iteratively learns (1) which words occur together (word distribution patterns), and (2) six distinct latent types of word distribution patterns called topics. The trained model is subsequently validated against a test dataset (Koch et al., 2014), and can thereafter be used on other datasets. Put simply, the *observed* word distributions in an epoch (Figure 2A,B, gray bars) are compared to each of the six *expected* word distributions for each topic (Figure 2B; colored bars) that were generated by the model. If the observed and expected distribution compare well, the probability of that topic is high, while if the distributions hardly overlap, the probability of that topic is low. The probabilities are normalized to a sum of 1 (Figure 2C). In this way, each 30 s epoch is represented as a mixture of six topic probabilities. Repeating this for every 30 s epoch in a recording results in a topic diagram (Figure 2D) where each vertical bin is a mixture of six topics, and the height of each stacked bar in that bin represents the probability of each topic. The most likely topic in each bin can be related–but does not equal–to one of the traditional sleep stages.

It should be noted that Figure 2 only shows the word distributions of δ, θ, α, and β power in one EEG channel, to promote clarity of the visualization. The actual LDA model uses all word distributions, i.e., of δ, θ, α, and β power of two EEG channels (C3-A2 and O1-A2); of the spectral power below 5 Hz of two EOG channels (EOGL-A2 and EOGR-A1); and of the cross-correlation values between the two EOG channels.

#### Characteristics of Topics

**Figure 3:**
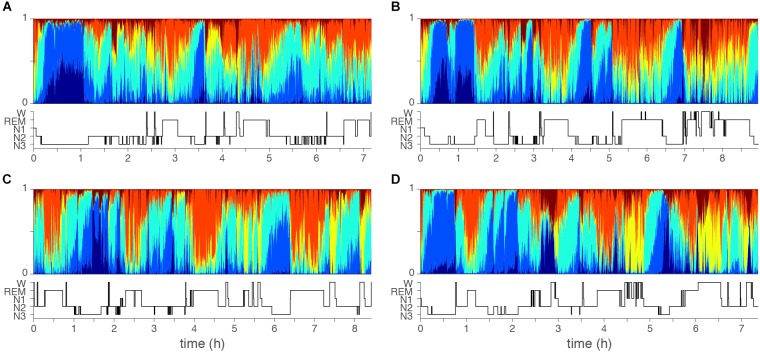
Examples of topic diagrams. Topic diagrams of two normal sleepers **(A,B)** and two insomniac patients **(C,D)**. Each 30 s sleep epoch is represented as a multi-colored vertical bin where the heigth of each stacked color presents the probability that the topic is present. For comparison, the manual scored hypnograms are presented below each diagram. Color codes range from dark blue (T1, mostly seen in deep N3 sleep) to red (T6, mostly seen in wakefulness). Orange (T5) is mostly seen during REM sleep. Note, however, that there is no one-to-one matching of topics and conventional top-down defined qualitative sleep stages. Subtle differences might be seen comparing the two controls **(A,B)** and two cases with insomnia **(C,D)**: In the epochs dominated by the light sleep related topic (yellow), the light sleep related topic is generally stronger (taller yellow bars) for the cases with insomnia as compared to controls.

Figure 3 shows four examples of topic diagrams and corresponding manually scored hypnograms. The topic diagram shows the mixture of vigilance states for each epoch. To facilitate understanding of the characteristics represented in each topic, we here describe the EEG signal in epochs with a particularly high probability of one of the six topics:

1. An epoch with a high probability of Topic 1 (T1, dark blue) has high and consistent delta and theta power. No eye movements are present, but EEG delta activity will be recorded from the EOGL-A2 and EOGR-A2 leads. An epoch rich in T1 will likely be labeled as stage N3 in conventional sleep scoring.
2. An epoch with a high probability of Topic 2 (T2, light blue) has high and consistent delta and theta power, intermediate alpha power and low beta power. No eye movements are present, but some EEG delta activity can be recorded from the EOGL-A2 and EOGR-A2 leads. An epoch rich in T2 will most likely be labeled as stage N3 in conventional sleep scoring.
3. An epoch with a high probability of Topic 3 (T3, turquoise) has intermediate delta, sigma, alpha and beta power in the EEG. No eye movements are present. An epoch rich in T3 will most likely be labeled as stage N2 in conventional sleep scoring.
4. An epoch with a high probability of Topic 4 (T4, yellow) has low delta and theta power, intermediate alpha power and intermediate to high beta power. Eye movements are likely. An epoch rich in T4 will most likely be labeled as stage N1 in conventional sleep scoring.
5. An epoch with a high probability of Topic 5 (T5, orange) has low delta power and low to intermediate theta, alpha and beta power. Eye movements are present. An epoch rich in T5 will most likely be labeled as REM sleep in conventional sleep scoring.
6. An epoch with a high probability of Topic 6 (T6, red) has low delta power, intermediate theta power, and high alpha and beta power. Eye movements are likely. An epoch rich in T6 will most likely be labeled as wakefulness in conventional sleep scoring.

### Topic Presence, Dominance, and Dynamics

For the topic diagram of each participant, we extracted several derived measures including topic dominance, and co-occurrence, as well as between-epoch transition probabilities as a measure of vigilance dynamics. First, the topic with the highest probability in an epoch was labeled as the dominant topic. Using this dominance label, we defined epochs as *stable* if they were part of at least three consecutive epochs with the same dominant topic. These stable epochs were selected to extract (1) the probabilities of the dominant topic (*dominance*) and (2) the probabilities of *co-occurence* of the remaining topics (normalized to 1 within each epoch). Finally, we computed *transition probability* as the number of transitions from a stable epoch dominated by one topic to an epoch dominated by any other topic normalized to the total number of possible transitions.

Case-control differences were evaluated for the following outcome measures:

1. The overall average probability of each of the six topics across the night.
2. The percentage of time spent in each of the six stable epoch types across the night.
3. The probability of the dominant topic in each of the six stable epoch types (*dominance)*.
4. The co-occurrance probabilities of each non-dominant topic in each of the six stable epoch types (*co-occurence*).
5. The probabilities to transition from a stable epoch dominated by one topic to an epoch dominated by each of the other topics.

### Spectral Analysis

In order to evaluate whether LDA modeling differs from traditional spectral analysis, we performed a fast-fourier spectral analysis of the same EEG channels that were used to compute the word distribution. Absolute spectral power was integrated across the standard clinical frequency bands for each 30-s epoch and averaged within each of the manually identified sleep stages.

### Statistical Analyses

Wilcoxon rank-sum tests evaluated group-differences in age and sleep characteristics including the total Insomnia Severity Index (ISI) score, the PSG-based measures, the standard spectral analysis measures, and three of the above LDA model-based measures: the overall average probability of each topic, the percentage of time spent in each of the stable epoch types, and the probability to transition from a stable epoch dominated by one topic to an epoch dominated by each of the other topics.

Because the number of stable epochs for each topic is different across participants, we did not calculate average values for the measures of *dominance* and *co-occurrence* across the night, but rather used mixed-effect general linear models to account for this variability. Accordingly, we evaluated group differences in the *dominance* and *co-occurrence* of each of the topics with group, age, and sex as fixed-effect factors, and a random intercept for each participant.

In addition to describing markers that distinguish ID patients from controls, we evaluated whether the significant markers were associated with the severity of insomnia complaints in the ID group. We used mixed-effects linear models to evaluate the association of the total score of the ISI as a measure of subjective insomnia complaints with topic presence, dominance, and co-occurrence. Age and sex were included in the models as covariates. We calculated partial correlation coefficients between the transition-probabilities and total score of the ISI, controlling for age and sex. In case the effect of the total ISI score was significant, we performed one additional mixed-effects linear regression analysis to elucidate which insomnia symptom could best explain the dependent variable by replacing the total ISI sum score with seven regressors indicating each individual ISI item.

The significance level was set to α = 0.05 in all cases. All analyses were performed using MATLAB, R2014a, The MathWorks, Inc., Natick, MA, United States.

## Results

Table 1 shows group averages and significance of differences between ID and controls for demographic characteristics, subjective insomnia severity, PSG measures, and LDA model-based measures. Participants with ID subjectively experienced significantly higher insomnia severity. PSG measures were comparable, except for significantly shorter total sleep duration in ID (controls: 421.7 ± 60.7 min, ID: 400.6 ± 59.3 min, Wilcoxon *W* = 4249, *z* = 2.18, *p* = 0.03).

### Overall Average Probability of Each of the Six Topics Across the Night

Table 1 reports the overall average probability of each of the six topics for controls and ID. The overall probabilities did not significantly differ between ID and controls for any topic.

### Percentage of Time Spent in Each of the Six Stable Epoch Types

Table 1 reports the percentage of time spent in each of the stable epoch types. No significant between-group differences were observed. Furthermore, there was no significant difference in the percentage of stable epochs irrespective of topic dominance between controls [mean (SD) = 56.08% (5.42%)] and ID [57.29% (6.59%); *W* = 3526, *z* = 1.20, *p* = 0.23].

### Dominance

**Figure 4:**
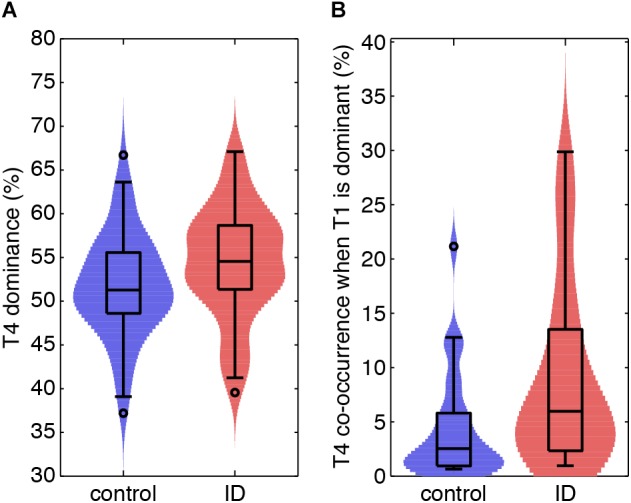
Light sleep-related EEG signatures are more abundant during light and deep sleep in ID than in controls. **(A)** Violin plots for each group (horizontal axis) of the probability of light sleep-related topic T4 when this is stable and dominant (vertical axis). **(B)** Normalized co-occurrence of topic T4 when deep sleep-related topic T1 is stable and dominant (vertical axis).

Table 2 reports the mean dominance of each stable epoch type for controls and ID. In stable epochs where the N1-related T4 was dominant, T4 was more dominant in ID than in controls (Figure 4A). The topic diagrams in Figure 2D, 3 shows T4 in yellow. These findings indicate that stable epochs of light sleep in ID contain more light sleep characteristics than stable epochs of light sleep in controls do.

**Table 2:** Increased light sleep dominance in ID.

Topic	control	ID	Δ	P
T1[%]	52.40 (49.69–55.11)	51.43 (48.81–54.05)	-1.28 (-5.28–2.72)	0.46
T2 [%]	67.81 (66.15–69.47)	67.46 (65.78–69.13)	-0.35 (-2.72–2.01)	0.48
T3 [%]	57.36 (56.43–58.29)	57.71 (56.67–58.75)	0.35 (-1.04–1.74)	0.68
**T4 [%]**	**52.59 (51.13–54.05)**	**55.28 (53.68–56.88)**	**2.74 (0.58–4.90)**	**0.03**
T5 [%]	61.11 (59.62–62.60)	61.44 (59.80–63.07)	0.31 (-1.90–2.52)	0.63
T6 [%]	51.75 (48.93–54.58)	50.09 (47.74–52.43)	-2.20 (-5.98–1.58)	0.33

*Estimated mean topic dominances defined as the mean probability of each topic when that topic is stable and 95%-confidence intervals for controls and subjects with Insomnia disorder (ID). Bold font-style indicates significant group differences*.

### Co-occurrence

Table 3 reports on the normalized co-occurrence of T2 to T6 in stable epochs dominated by T1 (N3-related topic). As T1 is related to (very) deep sleep, only 30/64 controls and 22/55 participants with ID expressed this stable epoch type (no between group differences in proportion of expression; Chi-square test, *p* = 0.45). For these stable epochs, we found a significantly higher co-occurrence of the N1-related T4 in ID (8.44%) as compared to controls [4.49%, *t*(551) = 2.32, *p* = 0.02; Figure 4B]. The co-occurrences of other topics in stable epochs dominated by T1 were not significantly different between ID and controls. These findings indicate that deep sleep in ID contains twice as much of the light sleep characteristics as compared to controls.

**Table 3:** Increased N1 sleep-related EEG signatures during deep sleep related periods in ID.

	Controls	ID	Δ	P
Co-occurrence of T2 [%]	33.48 (23.47–43.50)	21.15 (11.80–30.50)	-12.58 (-26.75–1.58)	0.10
Co-occurrence of T3 [%]	9.06 (5.60–12.52)	8.17 (5.62–10.72)	-0.81 (-5.33–3.70)	0.71
**Co-occurrence of T4 [%]**	**4.49 (2.70**-**6.29)**	**8.44 (5.43**-**11.46)**	**4.12 (0.79–7.44)**	**0.02**
Co-occurrence of T5 [%]	7.83 (4.63–11.03)	8.13 (5.39–10.86)	0.56 (-3.84–4.95)	0.84
Co-occurrence of T6 [%]	45.08 (36.71–53.46)	53.59 (43.57–63.62)	8.69 (-4.32–21.70)	0.20

*The normalized co-occurrence of topics T2-T6 in periods where the deep sleep-related topic T1 is dominant and stable. The values are reported as estimates, their 95th confidence intervals, and P-values obtained from linear mixed effect models with group, age and sex as fixed-effect factors, and a random intercept for each subject. Bold font-style indicates significant group differences*.

Similar tables for the other stable epoch types (stable epochs dominated by T2 to T6) are presented in Supplementary Table S1. None of the co-occurrences reported in the Supplementary Table S1 were found to differ between groups.

### Probabilities to Transit From Stable Epochs Dominated by One Topic to an Epoch Dominated by Each of the Other Topics

**Figure 5:**
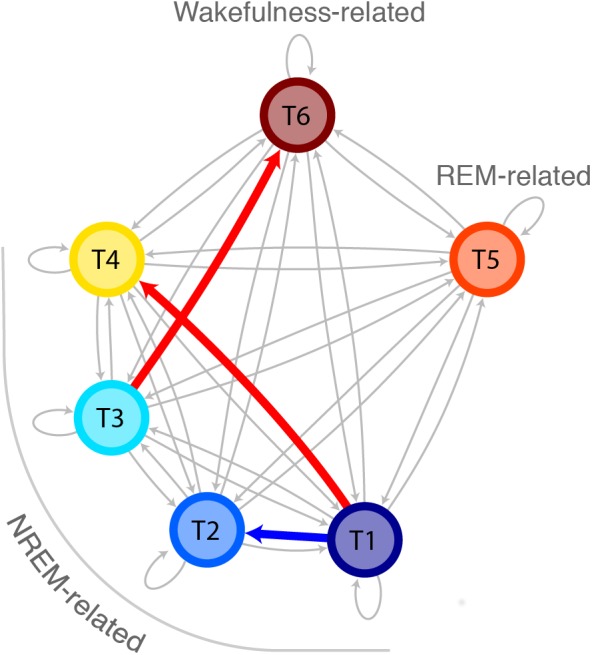
Increased probability for transitions from deep sleep to light sleep in ID as compared to controls. Markovian state diagram for topic transitions. The red arrow indicates a higher probability for participants with ID compared to controls, whereas the blue arrow indicates a lower probability for participants with ID compared to controls. The topics are indicated with colors and arranged in a way so the vertical axis denotes deepness of sleep.

The probabilities to switch from stable epochs of one topic to an epoch of any other topic are summarized in a Markovian state diagram (Figure 5). The transition probability from stable epochs dominated by the N3-related T1 to an epoch dominated by the N1-related T4 was about 8 times higher in participants with ID [*p*(T1→T4|ID) = 13.5%] compared to controls [*p*(T1→T4|C) = 1.7%; Wilcoxon *W* = 579, *z* = 2.36, *p* = 0.018]. In addition, the transition probability from the same stable epochs dominated by the N3-related T1 to an epoch dominated by another N3-related topic (T2) was about 6 times lower in participants with ID compared to controls [*p*(T1→T2|ID) = 2.6% compared to *p*(T1→T2|C) = 15.4%; Wilcoxon *W* = 394.5, *z* = -2.22, *p* = 0.026]. Finally, the transition probability from stable epochs dominated by the N2-related T3 to an epoch dominated by the wakefulness-related T6 was increased in participants with ID [*p*(T3→T6|ID) = 0.7%] compared to controls [*p*(T3→T6|C) = 0.5%; Wilcoxon *W* = 3406.5, *z* = 1.97, *p* = 0.048]. No other transitions were found to differ between groups. These findings indicate that compared to ID, controls have a higher tendency to switch from stable epochs dominated by one deep sleep related topic to epochs dominated by another deep sleep related topic, effectively remaining in deep sleep, whereas participants with ID have a higher tendency to switch to an epoch dominated by a light sleep related topic or from a stable epoch dominated by a N2-related topic to wakefulness.

### Spectral Analysis

We found that beta power in the occipital region during N1 sleep was significantly increased in participants with ID [mean(SD) = 93.3 μV (32.1 μV)] compared to controls [mean (SD) = 91.9 μV (90.2 μV); *W* = 3340, *z* = -2.20, *p* = 0.03).

### Associations With Subjective Severity of Insomnia Symptoms in People With Insomnia

Firstly, interindividual differences in the transition probabilities “T1→T4,” “T1→T2,” and “T3→T6,” and beta power in the occipital region during N1 sleep, were not significantly associated with total ISI scores (0.37 ≤ *p* ≤ 0.88). Secondly, whereas interindividual differences in total ISI scores could not explain T4 dominance (β [se] = -0.11 [0.17], *t*(1334) = -0.64, *p* = 0.52), we found that greater T4 co-occurrence in stable epochs dominated by T1 was significantly associated with higher total ISI scores (β [se] = 1.13 [0.23], *t*(192) = 4.85, *p* = 2.5 × 10^-6^). The follow-up analysis indicated that greater T4 co-occurrence in stable epochs dominated by T1 was associated with more difficulties staying asleep (β [se] = 4.17 [1.18], *t*(186) = 3.54, *p* = 5.0 × 10^-4^), with more worries/distress about sleep problems (β [se] = 5.06 [1.82], *t*(186) = 2.78, *p* = 0.006), and with less problems waking up too early (β [se] = -1.98 [0.94], *t*(186) = -2.11, *p* = 0.04). The other ISI items were not statistically significant (0.54 ≤ *p* ≤ 0.80).

## Discussion

We analyzed the sleep EEG of people with ID and controls with an automatic sleep analysis procedure that expresses every 30-s epoch as a mixture of vigilance states, here called topics, instead of a single sleep stage. Compared to controls, participants with ID had (1) a higher probability of a light sleep-related topic (T4) in stable epochs where light sleep is dominant, (2) a higher co-occurrence of the same light sleep related T4 in stable epochs where a deep sleep related topic (T1) is dominant. Additionally, (3) we found that people with ID had a higher probability to transition from a stable T1-dominant epoch to an epoch where a light sleep-related topic is dominant, while controls were more likely to transition to an epoch where another deep sleep related topic is dominant. Also, (4) we found that people with ID had a higher probability to transition from stable epochs of an N2-sleep related topic (T3) to an epoch dominated by the wakefulness-related topic (T6) as compared to controls. Finally, using a standard spectral analysis (5) we found that people with ID show increased beta power in the occipital region during N1 sleep as compared to controls.

Our findings indicate that people with ID have more EEG signatures typical of light sleep than controls do, both during epochs where light sleep prevails and during epochs where deep sleep prevails. Local sleep and wakefulness would imply simultaneous wake- and sleep EEG signatures, which is what these findings suggest. Together with our findings of increased transitions from deep sleep to light sleep in ID, the present study suggests that people with ID are hyperaroused even in their deepest sleep. Another notable finding is the lack of major differences in standard PSG measures, as a recent review has also concluded (Feige et al., 2013). In a similar vein, we did not find any group-differences in the average probability or time spent in any of the stable epochs dominated by one topic. Only total sleep time (TST) was statistically different. However, with a mere 20 min less TST in ID as compared to controls, this difference cannot explain the severe subjective insomnia complaints. Our novel approach detected that subtle markers of light sleep-related EEG features during deep sleep were associated with the severity of insomnia complaints. Greater co-occurence of light sleep signatures during deep sleep in insomnia were associated with more difficulties staying asleep, more worries/distress about sleep problems, and with less problems waking up too early. These findings indicate that although the differences in light sleep signatures during deep sleep between insomnia patients and controls are small, these subtle changes in sleep do correspond to subjective insomnia complaints. Another important finding is the opposite direction of associations with difficulties staying asleep and waking up too early. The occurrence of light sleep during deep sleep is positively associated with the subjective difficulty staying asleep but negatively with the subjective problem of waking up too early. The suggested differential association appeared in *post hoc* analyses and therefore requires replication.

Although this study utilized the patterns of power-spectral dynamics, our methodology goes beyond standard spectral analysis methods and analysis of specific micro-sleep phenomena. Our method is flexible and fully automated and is applicable for scoring and analyzing PSG data of ID patients. Nonetheless, our findings are in line with previous research that identified increased beta-power during NREM sleep in insomnia disorder (Freedman, 1986; Merica et al., 1998). Furthermore, we have previously implicated (Wei et al., 2017) an increased probability to transition from stage N2 to stage N1 or wakefulness in ID (based on manual scorings following the AASM scoring criteria); similarly, here we found increased transition probabilities from a N2-related T3 to a wakefulness-related T6, and from a deep sleep-related T1 to a light sleep-related T4 in ID.

A future aim could be to examine the temporal dynamics of the topics across the night, as the topic probabilities clearly show cyclic dynamics. Interestingly, for the REM-related topic (T5) we visually observed low probabilities for T5 at the start of the nights which increased with time. In the current study we did not compute an objective measure capturing these temporal dynamics because the current definition of sleep cycles depends on discrete classification of epochs. New measures of such dynamics need careful examination and validation against this golden standard.

### Importance for the Field and Limitations

The automatic sleep analysis procedure used in the current study has provided a more detailed representation of sleep as compared to the AASM standard. Expressing sleep epochs as a mixture of vigilance states allowed us to investigate sleep dynamics in a more refined way, and we identified alterations in sleep patterns of participants with ID that were otherwise not detectable by manual scoring methods. A future aim could be to develop a LDA model that utilizes all EEG leads from high-density EEG recordings. Such a development would extend the spatial dimension of the analysis. Another important consideration for PSG research in general is that the current analysis procedure is bounded by the 30 s epochs. For now, the LDA model is trained to compute topic probabilities for fixed sleep epochs with a constant length of 30 s. A future focus for automated sleep-stage classification could be to both allow indiscrete labeling (more stages at a time), and variable epoch lengths. It should, however, be noted that by allowing data-driven methods to define epoch boundaries and vigilance states, the method might yield sleep stages that are not defined by the AASM today. In addition, the time-scale at which the number of vigilance states is defined is itself a factor that defines the number of vigilance states that can be captured. Nonetheless, overcoming these challenges will increase our conceptual understanding of sleep, and allows for data-driven identification of sleep alterations in health and disease.

A limitation of the present study is that we did not correct for type-1 error-rate inflation. The significance of the results would not have survived a false discovery rate correction. The findings should be considered preliminary until replicated in an independent sample.

The current study has produced important findings that can benefit the understanding of the symptomology of ID in terms of sleep characteristics. In ID, light sleep-related sleep EEG power changes are more prominent not only in light sleep but also even in their deepest sleep.

## Ethics Statement

This study was carried out in accordance with the recommendations of the Central Committee on Research Involving Human Subjects with written informed consent from all subjects. All subjects gave written informed consent in accordance with the Declaration of Helsinki. The protocol was approved by the ethics committee of the Amsterdam University Medical Center, Amsterdam, Netherlands.

## Author Contributions

JC: study concept and design, development of topic model, and analysis and interpretation of data. RW: study concept and design, analysis and interpretation of data, and acquisition of data. YW: analysis and interpretation of data. JR and OL-K: acquisition and analysis of data. PJ: study concept and design, interpretation of data, and study supervision. EVS: study concept and design, interpretation of data, study supervision, and principal investigator. All the authors critically reviewed the manuscript.

## Conflict of Interest Statement

The authors declare that the research was conducted in the absence of any commercial or financial relationships that could be construed as a potential conflict of interest.

## Footnotes

1 https://www.sleepregistry.org

2 https://github.com/meegpipe/meegpipe

## Abbreviations

AASM: American Academy of Sleep Medicine
CAP: cyclic alternating pattern
EEG: electroencephalography
EOG: electrooculography
ERP: event-related potential
ID: insomnia disorder
ISI: insomnia severity index
LDA: latent Dirichlet allocation
NREM: non-rapid eye movement
PSG: polysomnography
REM: rapid eye movement
TST: total sleep time.
