# Supplementary material for: Data-Driven Analysis of EEG Reveals Concomitant Superficial Sleep During Deep Sleep in Insomnia Disorder
Source: Front Neurosci. 2019 Jul 9;13:598. doi: 10.3389/fnins.2019.00598 (PMC6629891; doi:10.3389/fnins.2019.00598)
Supplement: Supplementary file 1 [file Table_1.docx]

**Distribution of topics across AASM sleep stages**

In controls, a typical wake epoch was found to have high probabilities of topic T6 (31%), medium probabilities of T4 (29%), T1 (16%) and T5 (13%) and low probabilities of T3 (7%) and T2 (4%). A typical REM epoch showed a high probability of topic T5 (45%), medium probabilities of T4 (23%) and T3 (21%) and low probabilities of T2 (4%), T6 (3%) and T1 (3%). A typical N1 sleep epoch contained high probability of T4 (35%), medium probabilities of T5 (23%), T3 (19%) and T6 (13%) and low probabilities of T2 (5%) and T1 (4%). A typical N2 sleep epochs had high probability of T3 (46%), medium probabilities of T2 (18%), T5 (18%) and T4 (12%) and low probabilities of T6 (4%) and T1 (2%). Finally, a typical N3 sleep epoch was found to have high probability of T2 (60%), medium probabilities of T3 (19%) and T1 (12%) and low probabilities of T5 (6%), T6 (2%) and T4 (1%). Interestingly, in participants with ID, a similar distribution of topics across the different sleep stages was observed, indicating that the apparent distribution of topics across sleep is similar between ID and controls.

**Table S1: Values for the co-occurrences for the six different stable epoch types.**

The normalized co-occurrences of the non-dominant topics in periods where topic T1-T6 is dominant and stable. The values are reported as estimates, their 95th confidence intervals, and P-values obtained from linear mixed effect models with a random intercept for each subject.

|  | **Controls** | **ID** | **Δ** | **P** |
| --- | --- | --- | --- | --- |
| **T1 dominant and stable** | |  |  |  |
| Co-occurrence of T2 | 33.48 (23.47 – 43.50) | 21.15 (11.80 – 30.50) | -12.58 (-26.75 – 1.58) | 0.10 |
| Co-occurrence of T3 | 9.06 (5.60 – 12.52) | 8.17 (5.62 – 10.72) | -0.81 (-5.33 – 3.70) | 0.71 |
| **Co-occurrence of T4** | **4.49 (2.70 – 6.29)** | **8.44 (5.43 – 11.46)** | **4.12 (0.79 – 7.44)** | **0.02** |
| Co-occurrence of T5 | 7.83 (4.63 – 11.03) | 8.13 (5.39 – 10.86) | 0.56 (-3.84 – 4.95) | 0.84 |
| Co-occurrence of T6 | 45.08 (36.71 – 53.46) | 53.59 (43.57 – 63.62) | 8.69 (-4.32 – 21.70) | 0.20 |
| **T2 dominant and stable** | |  |  |  |
| Co-occurrence of T1 | 38.42 (35.10 – 41.74) | 37.57 (33.75 – 41.40) | -0.84 (-5.88 – 4.20) | 0.84 |
| Co-occurrence of T3 | 40.76 (38.17 – 43.35) | 41.32 (38.12 – 44.52) | 0.56 (-3.52 – 4.63) | 0.97 |
| Co-occurrence of T4 | 1.84 (1.65 – 2.02) | 2.23 (1.66 – 2.80) | 0.40 (-0.17 – 0.96) | 0.12 |
| Co-occurrence of T5 | 13.72 (10.74 – 16.71) | 14.11 (11.66 – 16.55) | 0.39 (-3.55 – 4.32) | 0.88 |
| Co-occurrence of T6 | 5.29 (4.22 – 6.35) | 4.76 (3.72 – 5.80) | -0.53 (-2.02 – 0.97) | 0.77 |
| **T3 dominant and stable** | |  |  |  |
| Co-occurrence of T1 | 3.08 (2.75 – 3.40) | 3.16 (2.67 – 3.65) | 0.08 (-0.49 – 0.66) | 0.70 |
| Co-occurrence of T2 | 37.10 (35.88 – 38.32) | 37.43 (36.00 – 38.85) | 0.32 (-1.55 – 2.19) | 0.84 |
| Co-occurrence of T4 | 20.18 (18.87 – 21.49) | 20.75 (19.46 – 22.04) | 0.56 (-1.28 – 2.41) | 0.45 |
| Co-occurrence of T5 | 31.27 (29.42 – 33.12) | 29.59 (27.61 – 31.57) | -1.68 (-4.39 – 1.04) | 0.24 |
| Co-occurrence of T6 | 8.39 (7.55 – 9.24) | 9.05 (8.25 – 9.84) | 0.64 (-0.53 – 1.82) | 0.34 |
| **T4 dominant and stable** | |  |  |  |
| Co-occurrence of T1 | 4.66 (3.32 – 5.99) | 3.65 (2.84 – 4.46) | -1.08 (-2.69 – 0.52) | 0.14 |
| Co-occurrence of T2 | 5.44 (4.47 – 6.40) | 5.19 (4.03 – 6.36) | -0.27 (-1.78 – 1.24) | 0.86 |
| Co-occurrence of T3 | 22.56 (19.97 – 25.15) | 23.41 (19.97 – 26.85) | 0.54 (-3.66 – 4.74) | 0.50 |
| Co-occurrence of T5 | 37.23 (34.17 – 40.29) | 36.25 (31.79 – 40.71) | -0.95 (-6.29 – 4.38) | 0.49 |
| Co-occurrence of T6 | 30.07 (26.39 – 33.75) | 31.74 (27.52 – 35.97) | 1.79 (-3.78 – 7.37) | 0.61 |
| **T5 dominant and stable** | |  |  |  |
| Co-occurrence of T1 | 8.93 (7.52 – 10.33) | 9.17 (7.58 – 10.77) | 0.24 (-1.88 – 2.36) | 0.79 |
| Co-occurrence of T2 | 6.90 (6.20 – 7.61) | 7.72 (6.69 – 8.75) | 0.84 (-0.39 – 2.07) | 0.09 |
| Co-occurrence of T3 | 33.93 (31.04 – 36.82) | 34.37 (31.61 – 37.12) | 0.44 (-3.59 – 4.46) | 0.59 |
| Co-occurrence of T4 | 46.95 (43.43 – 50.47) | 45.44 (41.75 – 49.12) | -1.51 (-6.62 – 3.59) | 0.35 |
| Co-occurrence of T6 | 3.30 (2.75 – 3.85) | 3.45 (2.89 – 4.01) | 0.15 (-0.63 – 0.97) | 0.82 |
| **T6 dominant and stable** | |  |  |  |
| Co-occurrence of T1 | 19.92 (16.42 – 23.42) | 19.34 (15.49 – 23.18) | -0.59 (-5.81 – 4.64) | 0.80 |
| Co-occurrence of T2 | 8.43 (6.05 – 10.80) | 9.21 (7.17 – 11.26) | 0.98 (-1.98 – 3.94) | 0.49 |
| Co-occurrence of T3 | 15.83 (12.86 – 18.80) | 17.83 (14.34 – 21.33) | 1.97 (-2.61 – 6.54) | 0.29 |
| Co-occurrence of T4 | 47.34 (41.46 – 53.23) | 47.88 (42.36 – 53.40) | 0.40 (-7.79 – 8.59) | 0.87 |
| Co-occurrence of T5 | 8.16 (5.42 – 10.91) | 6.08 (3.68 – 8.48) | -2.07 (-5.79 – 1.66) | 0.15 |
